# Effect of Vertical Shoot-Positioned, Scott-Henry, Geneva Double-Curtain, Arch-Cane, and Parral Training Systems on the Volatile Composition of Albariño Wines

**DOI:** 10.3390/molecules22091500

**Published:** 2017-09-08

**Authors:** Mar Vilanova, Zlatina Genisheva, Miguel Tubio, Katia Álvarez, Jose Ramón Lissarrague, José Maria Oliveira

**Affiliations:** 1 Misión Biológica de Galicia (CSIC), P.O. Box 28, 38080 Pontevedra, Spain; 2 CEB—Centre of Biological Engineering, University of Minho, 4710-057 Braga, Portugal; zlatina@deb.uminho.pt (Z.G.); jmoliveira@deb.uminho.pt (J.M.O.); 3 Martín Códax Winery, Vilariño, 36630 Cambados, Spain; mtubio@martincodax.com (M.T.); kalvarez@martincodax.com (K.Á.); 4 Escuela Técnica Superior de Ingenieros Agrónomos, Department of Agricultural Production, Universidad Politécnica de Madrid, 28040 Madrid, Spain; joseramon.lissarrague@upm.es

**Keywords:** wine quality, aroma, training systems, Albariño

## Abstract

Viticultural practices influence both grape and wine quality. The influence of training systems on volatile composition was investigated for Albariño wine from Rías Baixas AOC in Northwest Spain. The odoriferous contribution of the compounds to the wine aroma was also studied. Volatile compounds belonging to ten groups (alcohols, C_6_-compounds, ethyl esters, acetates, terpenols, C_13_-norisoprenoids, volatile phenols, volatile fatty acids, lactones and carbonyl compounds) were determined in Albariño wines from different training systems, Vertical Shoot-Positioned (VSP), Scott-Henry (SH), Geneva Double-Curtain (GDC), Arch-Cane (AC), and Parral (P) during 2010 and 2011 vintages. Wines from GDC showed the highest total volatile composition with the highest concentrations of alcohols, ethyl esters, fatty acids, and lactones families. However, the highest levels of terpenes and C_13_-norisoprenoids were quantified in the SH system. A fruitier aroma was observed in Albariño wines from GDC when odor activity values were calculated.

## 1. Introduction

Aroma is an important quality factor in white wines, and several compounds contribute to their complexity and belong to diverse groups such as alcohols, aldehydes, ketones, esters, fatty acids, terpenes, C_13_-norisoprenoids, etc. [1,2,3].

The volatile composition of wine can be influenced by several factors, such as grape variety, the degree of ripeness, growing climate, canopy management, fermentation conditions and winemaking, and ageing practices [4,5,6,7,8,9].

From viticultural practices, the use of several training systems, which can influence microclimate conditions, involves changes in different conditions affecting the content of vine metabolites, thus contributing to the final wine composition.

Several studies on training systems have demonstrated the effects on grape and wine composition [10,11,12,13,14,15,16,17]. Geneva Double-Curtain (GDC) showed the highest concentrations on Viognier volatile composition, as GDC wines are generally more fruity and floral [13]. GDC training systems consist in parallel bilateral cordons with spurs retained along these cordons. Vines in the row are alternated to the left or right cordon wire to give the double curtain effect [15]. GDC amended for better fruit maturation because it reduces the shade and enhances the grape and wine qualities [18]. Training systems such as vertical shoot positioning (VSP) were found to significantly affect the aroma profile of Cabernet-Sauvignon must [10] and produced the highest amount of geraniol and C_6_ aldehydes in Traminette grapes [12]. VSP is used to train the vine shoots upward in a vertical curtain, creating a fruit zone below. Its advantages over other training systems include the possibility of machine harvesting, good air circulation, and optimal light exposure [13,19]. In general, wines produced from training systems with divided canopies could increase grape and wine quality due to an increase canopy volume and the fact that a greater percentage of the leaf surface area can be exposed [13]. In the Scott-Henry (SH) training system the shoots are separated and trained in two vertical curtains upward and downward, increasing the fruiting area and allowing more sun penetration. It is suitable for mechanical harvesting and improves wine quality [18]. The arch cane (AC) training system, also known as Pendelbogen, trains upward from the ground. By bending the shoots in an arch, this training system achieves more fruit bearing shoots, thereby higher yields [11]. However, the higher quantity of grapes can reduce the ripeness level. The Parral training system, also known as Parron, has high yields and captures the most sunlight. Because of its high cost to construct and maintain, the Parral system is not often used. When using this system, the canopy is dense and the ventilation of air is difficult, which can cause the development of grape diseases [18]. Although there are many studies on the effect of the training system on fruit composition, some research has shown that the training system does not influence fruit or wine composition.

Albariño is the most important white cultivar grown in Galicia, in Northwest Spain. Both the grapes and wines have been the focus of several studies [20,21,22,23,24]. Albariño grapes are characterized by high floral intensity, fruity descriptors, and free monoterpenes, which are responsible for these floral notes [20,25,26]. Several studies have shown that the main compounds contributing to the flavour of Albariño wines, through instrumental analysis, were those related to fruity aromas, such as ethyl esters and acetates, and floral aromas, caused by monoterpenes [24]. Through sensory analysis, citric, flowers, fruit, ripe fruit, apple, and tropical were characteristic descriptors of Albariño wines from Rías Baixas AOC [24].

Parral is a traditional horizontal training system applied in Galicia that together with VSP are the most common training systems in this wine-growing area; however, several experiences with other less traditional systems are being studied. Despite the importance of Albariño as Spanish white wine, there is little information on the optimization of cultural practices on their quality. The aim of this work was to evaluate the influence of five different training systems on the volatile composition of Albariño wines.

## 2. Materials and Methods

### 2.1. Vineyard Locations and Weather Conditions

*Vitis vinifera* L cv. Albariño vines were grown on different training systems in an experimental vineyard “Pe Redondo” of Martín Códax Winery located in Rías Baixas AOC from Galicia, NW Spain (42°30′21.11″ North, 8°43′32.55″ West, 150 m altitude). The area has maritime Mediterranean climate, humid, with mild winters, and warm summers. Average annual temperature is around 14.5 °C; and average rainfall, 1400 mm to 1500 mm. The most representative soils of the area are Haplumbrept, have an acidic pH, are rich in organic matter, and have aloamy texture. Specific climatic conditions by year are shown in Table 1.

The five training systems were: Vertical Shoot-Positioned (VSP), Scott-Henry (SH), Geneva Double-Curtain (GDC), Parral (P) and Arch-Cane (AC), also called Pendelbogen or Capo Volto. Vines were grafted to 420-A rootstock and planted in 2003. The experiment design involved the five different training systems in a randomized complete block design with four replications across four blocks. During ripening, the vineyards were drip irrigated, with annual water dosages ranging from 900 m^3^/ha to 1200 m^3^/ha.

### 2.2. Yield Components and Grape Composition

At harvest, yield data were collected by hand-harvesting fruit from vines of each replicate at similar ripening stage (°*Brix* from 20.3 to 21.4, Table 2) during two consecutive vintages, 2010 and 2011. Bunches and yield per shoot and per vine were recorded, from which average bunch weights and berries per bunch were determined. Berry samples (100 per replicate) were used for determination of berry weight. Each experiment was carried out in triplicate for berry and cluster samples from five training systems.

A total of 300 berries were collected for each replicate. Total soluble solids (°*Brix*) were analyzed by refractometry and the grapes were immediately frozen and stored at −20 °C until further analyses.

### 2.3. Fermentations

The Albariño white wines were made in the Martín Códax Winery. White wines were produced in 100 L inox tanks. Before fermentation, sulfur dioxide (5 g/hL) was added to the musts. The wines were made using standard winemaking practices. The fermentation was conducted by yeast strain *Saccharomyces bayanus* CHP AZ 3 Oeno at 18 °C. After fermentation, sulfur dioxide (4 g/hL) was added and the wines were filtered and transferred to 0.75 L bottles. The bottles were stopped with a cork and stored at 16 °C until analysis.

Alcoholic strength, by volume (*AS*), total acidity (*TA*), volatile acidity (*VA*), pH, and tartaric acid, and malic acid mass concentrations were determined after alcoholic fermentation. For each vintage, wines were analyzed approximately two months after the end of the winemaking. All analyses were performed in triplicate and were determined using a Foss WineScan™ FT120, as described by the manufacturer (Foss, Hillerød, Denmark). Foss WineScan was calibrated by WinISI calibration software (Foss, Warrington, UK) and by comparison with OIV official methods [27].

### 2.4. Extraction, Identification and Quantification of Volatile Compounds

Volatile compounds were analyzed by gas chromatography–mass spectrometry (GC-MS) after extraction of 8 mL of wine with 400 mL of dichloromethane, spiked with 3.28 μg of 4-nonanol (Internal Standard), according to the methodology proposed by Oliveira et al. [7]. A gas chromatograph Varian 3800 with a 1079 injector and an ion-trap mass spectrometer Varian Saturn 2000 was used. A 1 μL injection was made in splitless mode (30 s) in a Varian Factor Four VF-Wax ms column (30 m × 0.15 mm; 0.15 μm film thickness). The carrier gas was helium UltraPlus 5× (Praxair) at a constant flow rate of 1.3 mL/min. The detector was set to electronic impact mode with an ionization energy of 70 eV, a mass acquisition range from 35 *m*/*z* to 260 *m*/*z*, and an acquisition interval of 610 ms. The oven temperature was initially set to 60 °C for 2 min and then raised from 60 °C to 234 °C at a rate of 3 °C/min, raised from 234 °C to 250 °C at 10 °C/min, and finally maintained at 250 °C for 10 min. The temperature of the injector was maintained at 250 °C during the analysis, and the split flow was maintained at 30 mL/min. The identification of compounds was performed using the software MS Workstation version 6.9 (Varian, Inc. Walnut Creek, CA, USA) by comparing their mass spectra and retention indices with those of pure standard compounds. The compounds were quantified in terms of 4-nonanol equivalents only. Pure standard compounds were purchased from Sigma-Aldrich (Darmstadt, Germany) with purity higher than 98%.

### 2.5. Odor Activity Values

The odor activity value (*OAV*) was calculated for each quantified compound as the ratio between the concentration of an individual compound and the perception threshold in the same matrix found in the literature [1,28,29,30].

### 2.6. Analysis of the Data

All data were analyzed using the software XLStat-Pro (Addinsoft, Paris, France, 2011). Data were analyzed using a general linear model to test significant differences among training systems by analysis of variance (ANOVA). Fisher’s Least Significant Difference (LSD) means comparison test (*p* < 0.05) was performed. Principal Component Analysis (PCA) was used on wine volatile composition to discriminate among training systems according these data.

## 3. Results and Discussion

### 3.1. Yield Components and Wine Chemical Composition

Components of yield, as the mean of 2010 and 2011 vintages, of five training systems are presented in Table 2. The training system did not show any effect on grape ripening. Significant differences regarding shoot density were noted, with Parral and GDC differing by up to 30%. Parral was the training system with a higher crop yield, followed by GDC, VSP, SH, and AC. Berry weight at harvest did not vary among systems, but the Parral system had a greatest cluster weight because the highest number of berries per cluster, followed by GDC system. Generally, increases in yield due to training systems tended to result from increases in cluster per vine [15]. However, in our study increases in yield were due to increases in berries per cluster and therefore cluster weight, where the Parral, followed by the GDC, training systems showed the highest values.

The values (mean and standard deviation from 2010 and 2011 vintages) of classical chemical parameters of Albariño wines are shown in Table 3. The training system did not show any effect on chemical composition of the wines. In general, berry maturity was not altered by training system in the Cabernet sauvignon from the wet region of China [31,32]. Similar results were observed in cv. Viognier when it was compared to VSP, Smart Dyson, and GDC training systems, but Fragasso et al. [16] observed higher Primitivo grape ripeness when it was grown with the Lira training system versus bilateral Guyot and four rays.

### 3.2. Volatile Composition of Albariño Wines

A total of 35 compounds were identified and quantified in Albariño wines from different training systems using GC-MS, classified into nine groups (alcohols, C_6_-compounds, ethyl esters, acetates, terpenes+C_13_-norisoprenoids, volatile phenols, fatty acids, lactones and carbonyl compounds) according to their functional groups and metabolic formation (Table 4 and Table 5).

**Figure 1:**
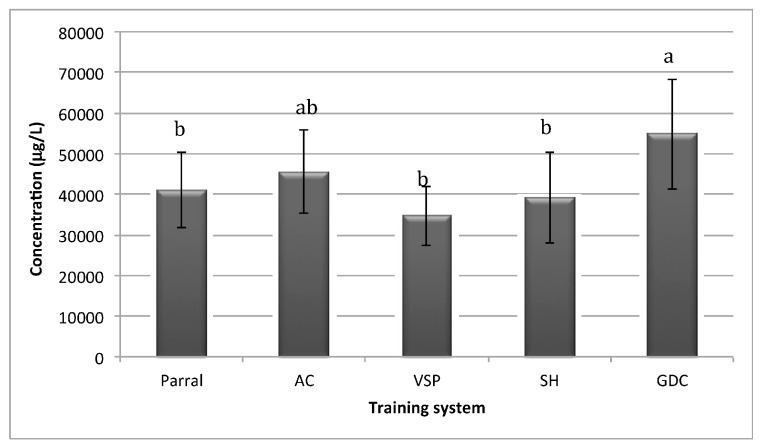
Total volatile concentration of Albariño wines from different training systems (mean and *SD*). Parral; AC: Arch-Cane; VSP: Vertical Shoot-Positioned; SH: Scott-Henry and GDC: Geneva Double-Curtain Training systems. Different letters show significant differences among wines by Fisher’s least significant difference (LSD) at *p* < 0.05.

Figure 1 shows the total concentration of volatile compounds of Albariño wines from different training systems. According to the quantitative data shown in Figure 1, total concentration of volatile compounds ranged from 34.42 mg/L to 54.81 mg/L. Wines from the GDC training system had the highest concentration of total volatile compounds followed by AC, while VSP had the lowest. In terms of total volatile composition, the GDC training was found to be statistically different from the other training systems (*p* < 0.05). These results suggest that the chosen training system could affect the wine volatile composition.

In order to analyze these differences in total volatile composition for wines from different training systems, a comparison of each chemical group was performed. The majority of volatiles were alcohols, ethyl esters, acetates, and fatty acids. The ANOVA analysis demonstrated that only the groups of alcohols, ethyl esters, terpenes+C_13_-norisoprenoids, and lactones exhibited statistical differences amongst wines from different training systems (Table 4). The GDC training system had the significantly highest total concentration of alcohols, ethyl esters, and lactones (*p <* 0.05), while SH had the highest concentration of terpenes and C_13_-norisoprenoids.

Albariño wines are known for being rich in ethyl esters, creating a fruity characteristic [22]. Ethyl esters are formed during the process of fermentation and are related to the lipid metabolism, except diethyl succinate and diethyl malate. Ethyl esters are among the most important compounds in wine [33] and their formation depends on fermentation practices, yeast strain, temperature of fermentation, aeration, and solids content of the must [34]. In the present study, all the wines were made with grapes harvested to the same ripening stages and under the same fermentation condition, so the differences between the samples can probably be explained by the different yield components caused by the training systems. Six ethyl esters were identified and quantified in wine samples and the total concentrations were between 1558 μg/L and 2569 μg/L. Wines from GDC showed the highest content of ethyl esters and SH had the lowest. Among ethyl esters, the most abundant were ethyl hexanoate, ethyl butanoate (both with fruity and strawberry aromas), and ethyl octanoate (apple, sweetish aroma). Ethyl hexanoate, ethyl butanoate, and diethyl malate were found in significantly high concentrations for wines from the GDC training system (*p* < 0.05) (Table 5).

Higher alcohols are synthetized by yeast during alcoholic fermentation [34]. In this study, nine alcohols were identified and quantified with total concentration from 18.95 mg/L and 33.20 mg/L. Wines from GDC showed the highest content of alcohols and VSP had the lowest (Table 5). Three compounds, 2+3-methyl-1-butanol and 2-phenylethanol, had the highest concentrations in all wines, but only 2-phenylethanol showed significant differences among training systems with the highest concentration in the GDC system (*p* < 0.05). Moreover, one other alcohol, 3-methyl-1-pentanol, was shown to be the significantly highest in the GDC system.

In terms of total concentration of terpenes together with C_13_-norisoprenoids, the SH training system showed the highest value at 69 μg/L, while the lowest concentration was for the SVP system at 38 μg/L. Three terpenes (linalool, hotrienol and α-terpineol) and one C_13_-norisoprenoid (β-damascenone) were identified and quantified in wines, but only hotrienol showed significant differences among training samples, with the highest value being obtained for SH (*p* < 0.05). Linalool and α-terpineol exhibited higher concentrations for SH, as did β-damascenone for GDC. Terpenes and C_13_-norisoprenoides are varietal compounds responsible for the aromatic quality of the wine, bringing fruity, citrus, and floral nuances [33,34,35].

Several viticultural parameters, including sunlight exposure, have been shown to influence terpene and norisoprenoid concentrations [12,36,37,38] and lower fruit temperature favors the accumulation of volatile compounds with lower molecular weights and boiling points [13]. Concentrations of TDN, vitispirane, hydroxy-TDN, ionols, actinidols, grasshopper ketone, and vomifoliol were significantly higher in sun-exposed Chenin blanc and white Riesling grapes compared to shaded grapes of the same varieties. However, β-damascenone concentrations did not appear to be influenced by sunlight levels [36]. The Scott-Henry training system generates a vertically divided canopy increasing the effective canopy surface area and reducing its density. Canopy division also improves light exposure [13].

The lactones group, represented by only one compound, γ-butyrolactone, showed concentrations between 91 μg/L and 253 μg/L. The highest value was reached in the GDC training system (*p* < 0.05). γ-butyrolactone is a volatile compound found in wine and produced during fermentation and is responsible for a cheesy aroma [29]. This compound reached higher concentrations (>3.7 mg/L) in Loureira and Albariño wines from Galicia [33].

Other individual compounds showed significant differences among wines from different training systems. From the acetates, 2-phenylethyl acetate reached concentrations between 331.5 μg/L and 499.6 μg/L (Table 5). The highest concentration was observed in wine from the GDC system. From the fatty acids, two compounds, hexanoic acid and octanoic acid, which creates a sweaty and cheese aroma, showed significant differences among the wines. The highest concentrations were also found in the GDC training system.

In summary, of the 35 volatile compounds identified and quantified in this study, ten showed significant differences among wines from five different training systems, where the highest concentrations were observed in the GDC system. GDC reached higher yields, however, the quality of the wine did not change. Yields can be increased with the appropriate training system without detrimentally impacting the fruit quality [15]. GDC increased the fruiting areas and has a split canopy that allows more sun penetration. Canopy division has been reported to increase canopy volume and surface area compared to non-divided VSP systems in previous studies [39]. Zoecklein et al. [13] also showed that, despite a significantly increased crop yield in cv. Viognier, the fruit glycosides and free volatiles were greater in the divided canopies with Smart Dyson and GDC, than in VSP.

### 3.3. Odor Active Values for Albariño Wines

The odor activity values (*OAV*) of each individual compound were calculated. *OAV* is used as an indicator for the potential sensorial contribution of each compound to the overall aroma of the wine. Compounds with *OAV* > 1 are considered as having a dynamic contribution to the wine aroma [40]. However, the wine aroma is a result of the synergic effect and interactions of all present chemical compounds. Table 6 lists the odorant activity values (*OAV*), odor threshold, and odor descriptor for the aromatic compounds where *OAV* > 1. From the 35 identified and quantified volatile compounds, 14 had an *OAV* > 1. The compounds that showed the highest *OAV* were β-damascenone (*OAV* > 151) and isoamyl acetate (*OAV* > 74), both with fruity characteristics of baked apple and banana, respectively. These results align with the findings of Falqué et al. [25], demonstrating that isoamyl acetate had high *OAV* and a positive effect on the aroma of Albariño wines. Vilanova et al. [33] found the same results when studying the volatile composition of white wines from Galicia. In this study, higher *OAV* values were reached by β-damascenone and isoamyl acetate for Albariño wines.

From the ethyl esters, the content of ethyl hexanoate was higher than their odor threshold in all wines analyzed, with the highest value found in GDC, with an *OAV* of 12 (Table 6). Other ethyl esters, including ethyl butanoate and ethyl octanoate, also showed values above their odor threshold. In both compounds, the highest *OAV* was exhibited for the GDC system, at 2.8 and 1.1, respectively (Table 6). These results are in accordance with those published by Zoecklein et al. [13], who concluded that wines from the GDC system had stronger fruity characteristics.

Among the higher alcohols, 2-phenylethanol contributed positively to the wine aroma with a rose aroma [1]. In our study, an *OAV* > 1 was found for GDC, AC, and SH wines, with the highest value (1.5) obtained from the GDC system (Table 6).

Five volatile acids showed *OAV* values > 1, where octanoic, hexanoic, and decanoic acids had the highest value in the GDC system. Falqué et al. [25] also found that octanoic and hexanoic acids were the most important acids in the Albariño wines. Vilanova et al. [33] showed hexanoic acid having a high *OAV* in Albariño wines from Galicia.

Vinylphenol, with a total concentration above 100 μg/L, is known to be found in large quantities in Albariño wines [23,25]. In the present study, 4-vinylguaiacol had an *OAV* between 1.4 for VSP and 2.9 for GDC. The major positive *OAV* units were found in the GDC training system, at 381.2, followed by Parral at 326.5, Arch-Cane at 309.9, and Scott Henry at 307.2; the smallest value was found in the VSP training system at 293.7.

### 3.4. Differentiation of Training Systems in Basis to OAV > 1

**Figure 2:**
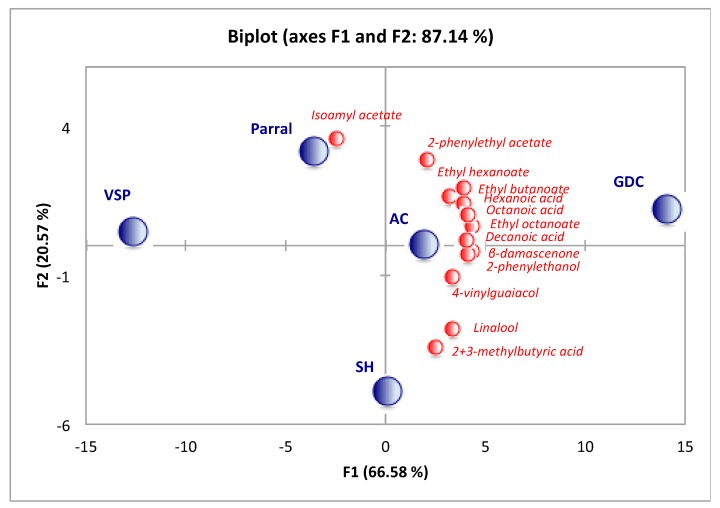
Principal Component Analysis (PCA) by *OAV* > 1 in Albariño wines from different training systems Vertical Shoot-Positioned (VSP), Scott-Henry (SH), Geneva Double-Curtain (GDC), Arch-Cane (AC) and Parral.

PCA was performed on the volatile composition to visualize the differentiation of wines on the basis of training system (Figure 2). The PCA was conducted on volatile compounds with an *OAV* > 1 (Table 6) of Albariño wines from different training systems.

The first two principal components accounted for 87.14% of the total variance, 66.58% and 20.57%, respectively. PCA exhibited good discrimination amongst Albariño wines. The PCA shows the distribution of wines according to training system, where most families of volatile compounds contributed to wines from GDC training system. Therefore, grapes produced with the GDC training system will result in fruitier wines. Linalool and 2+3-methylbutyric acids characterized the SH training system and isoamyl acetate, the Parral system. Nevertheless, VSP was distinguished from the rest training systems, and it was not related to any group of compounds.

## 4. Conclusions

The effects of five different training systems, GDC, AC, SH, VSP, and Parral, on Albariño wine volatiles were evaluated in Rías Baixas AOC. The Parral and GDC systems showed the highest yields because they produced the highest number of berries per cluster and therefore higher cluster weights were found. The volatile compounds results showed that Albariño wines from the GDC training system had the highest total concentration, while VSP showed the lowest concentrations. Comparing the subtotal of each chemical group of volatiles, wines from GDC had higher contents of alcohols, ethyl esters, and γ-butyrolactone, and the SH training system was associated with the highest concentrations of terpenes and C_13_-norisoprenoids. The PCA on odor activity values demonstrated fruity aromas characterized Albariño wines from the GDG system. In summary, this differentiation of the training systems on the basis of volatile compounds means that the final wines will have different aroma nuances. In accordance with other reports, the GDC system had higher fruity and floral aromas compared to other systems. In this context, similar studies are important to help to better understand and to improve or differentiate produced wines.

## Figures and Tables

**Table 1:** Meteorological parameters for the growing season April–September (2010 and 2011).

Climatic Conditions	Year (April–September)	
	2010	2011
Mean Temperature (°C)	17.1	17.3
Maximum Temperature (°C)	28.9	28.8
Minimum Temperature (°C)	9.7	10.6
Relative humidity (%)	71.9	73.7
Sunlight duration (h)	1925.6	1892.2
Mean daily irradiation (10 kJ/(m^2^ day))	2066.6	2079.3
Insolation (%)	65.6	64.4
Wind Speed (km/h)	19.8	18.6
Rain (L/m^2^)	343.4	311.4

**Table 2:** Must soluble solids (SST) and yield components at harvest from five training systems (mean 2010 and 2011).

Must SST and Yield components	Training System				
	Parral	AC	VSP	SH	GDC
Soluble solids, °*Brix*	21.4 ± 0.6	21.3 ± 1.0	20.3 ± 1.6	21.1 ± 0.1	21.3 ± 0.7
Yield (kg/ha)	19 528.5 a	12 993.6 c	17 537.9 b	16 163.8 b	18 488.3 ab
Shoot density (m^–2^)	9.1 a	6.7 c	8.3 ab	8.0 b	6.4 c
Cluster/shoot	1.9 b	1.7 b	1.7 b	1.8 b	2.3 a
Cluster weight (g)	151.2 a	111.2 c	120.8 c	123.0 bc	140.0 ab
Berries/cluster	123.9 a	96.7 c	103.3 c	106.0 bc	119.7 ab
Berry weight	1.2	1.2	1.2	1.2	1.2

Parral; AC: Arch-Cane; VSP: Vertical Shoot-Positioned; SH: Scott-Henry and GDC: Geneva Double-Curtain Training systems. Different letters show significant differences among wines by Fisher’s least significant difference (LSD) at *p* < 0.05.

**Table 3:** Effects of training system on wine chemical composition (mean 2010–2011 and standard deviation).

Chemical Composition	Parral	AC	VSP	SH	GDC
pH	3.0 ± 0.6	3.1 ± 0.1	3.0 ± 0.1	3.0 ± 0.0	3.1 ± 0.0
Volatile acidity (g/L)	0.4 ± 0.1	0.4 ± 0.1	0.4 ± 0.1	0.4 ± 0.0	0.4 ± 0.1
Total acidity (g/L)	9.7 ± 0.6	9.6 ± 0.6	9.9 ± 0.9	9.4 ± 1.1	9.4 ± 0.6
Tartaric acid (g/L)	5.7 ± 0.6	5.2 ± 0.2	5.6 ± 0.8	5.3 ± 0.9	5.4 ± 0.1
Malic acid (g/L)	4.2 ± 0.5	3.9 ± 0.5	4.1 ± 0.7	3.7 ± 0.5	3.6 ± 0.3
Ethanol (% *v*/*v*)	12.7 ± 0.4	12.3 ± 0.7	11.5 ± 1.0	12.7 ± 0.0	12.9 ± 0.3

Parral; AC: Arch-Cane; VSP: Vertical Shoot-Positioned; SH: Scott-Henry and GDC: Geneva Double-Curtain Training systems.

**Table 4:** Volatile composition (μg/L), by families, of Albariño wines from different training systems (mean 2010–2011 and *SD*).

Volatile Families	Parral		AC		VSP		SH		GDC	
	Mean	SD	Mean	SD	Mean	SD	Mean	SD	Mean	SD
Alcohols	22 104.6 b	6338.8	25 784.2 ab	8116.9	18 951.3 b	5110.6	22 345.3 b	7222.1	33 205.2 a	9852.5
C_6_–alcohols	249.0	60.5	302.9	58.7	266.9	73.0	251.4	70.1	258.6	68.4
Ethyl esters	1752.0 b	644.7	1799.9 b	505.7	1615.3 b	999.2	1558.6 b	375.3	2569.2 a	1404.2
Acetates	4069,2	560.4	3201.7	890.1	3728.1	866.3	2678.1	789.6	3199.2	1080.7
Terpenes+C_13_–norisoprenoids	46.4 bc	23.8	57.5 ab	10.7	38.0 c	13.6	69.0 a	25.4	67.9 bc	12.0
Volatile phenols	179.8	84.0	211.0	47.3	107.7	43.4	189.4	80.8	187.9	56.7
Fatty acids	12 479.5 ab	2836.2	14 040.4 a	3011.2	9832.3 b	2276.8	11 850.6 ab	4382.9	15 183.6 a	4193.6
Lactones	162.3 b	47.0	161.5 b	86.6	91.2 b	38.1	160.9 b	50.8	253.0 a	65.6

Parral; AC: Arch-Cane; VSP: Vertical Shoot-Positioned; SH: Scott-Henry and GDC: Geneva Double-Curtain Training systems. Different letters show significant differences among wines by Fisher’s least significant difference (LSD) at *p* < 0.05.

**Table 5:** Concentration (μg/L) of volatile compounds of Albariño wines from different training systems (mean 2010–2011 and SD).

Volatile Compounds	Parral		AC		VSP		SH		GDC	
	Mean	SD	Mean	SD	Mean	SD	Mean	SD	Mean	SD
***Alcohols***										
1-propanol	101.8	38.1	149.9	38.2	411.7	406.0	120.9	43.4	126.4	69.3
2-methyl-1-propanol	429.4	152.8	414.0	102.0	360.4	209.5	371.5	156.5	498.1	175.8
1-butanol	15,6	9.4	15.4	4.8	17.9	5.2	12.3	3.8	16.9	6.9
2+3-methyl-1-butanol	13 549.6	4537.4	14 730.9	3754.1	11 864.0	3095.6	11 708.8	3205.8	17 147.5	5505.1
2-methyl-1-pentanol	6.5	4.8	14.7	0.8	4.5	3.9	7.1	6.3	15.3	7.0
3-methyl-1-pentanol	15.7 b	5.8	16.9 b	9.3	8.7 b	3.0	14.2 b	6.0	24.5 a	6.5
2-phenylethanol	7968.7 b	1585.8	10 423.4 b	4199.7	6257.2 b	1339.4	10 090.1 b	3792.7	15 346.2 a	4072.0
3-methylthiopropanol	17.7	4.6	19.1	7.9	26.9	47.9	20.5	7.6	30.3	9.8
***C_6_–alcohols***										
1-hexanol	218.7	39.7	268.2	41.8	240.0	59.6	223.0	50.9	225.6	55.0
*E*-3-hexen-1-ol	12.2	10.6	9.2	6.9	8.5	6.9	9.5	7.9	9.7	5.2
*Z*-3-hexen-1-ol	18.1	10.3	25.5	10.1	18.4	6.6	18.9	11.3	23.3	8.3
***Ethyl esters***										
Ethyl butanoate	170.9 b	37.9	164.0 b	36.4	179.4 b	65.8	140.1 b	21.6	347.8 a	230.8
Ethyl hexanoate	631.9 abc	130.2	655.3 ab	49.5	518.8 c	108.9	555.6 bc	86.2	742.2 a	99.3
Ethyl octanoate	490.3	346.7	536.4	200.4	423.8	197.0	492.2	141.0	635.4	243.0
Ethyl decanoate	82.9	29.6	81.5	12.9	44.9	7.6	78.5	30.0	74.8	18.0
Diethyl succinate	105.2	25.7	86.6	56.3	297.5	563.0	95.3	30.5	429.9	730.1
Diethyl malate	270.7 abc	74.6	276.0 ab	150.2	150.9 c	56.9	196.8 bc	66.1	339.1 a	82.8
***Acetates***										
Isoamyl acetate	3458.8	482.8	2759.5	783.5	3230.8	738.2	2227.0	678.4	2598.6	927.5
Hexyl acetate	117.0	19.4	110.7	27.6	144.1	60.0	104.5	29.5	101.0	21.7
2-phenylethyl acetate	493.4 a	58.3	331.5 b	79.0	353.2 b	68.2	346.5 b	81.7	499.6 a	131.5
***Terpenes+C_13_–norisoprenoids***										
Linalool	18.7	18.1	25.7	6.0	18.7	7.8	33.7	11.3	33.1	6.0
hotrienol	18.2 a	2.3	20.6 a	4.1	10.5 b	5.0	22.4 a	7.7	22.0 a	4.5
α-terpineol	1.2	0.1	2.7	0.4	1.2	0.0	3.4	1.6	0.8	0.1
β-damascenone	8.4	3.4	8.5	0.2	7.6	0.8	9.5	4.8	12.0	1.5
***Volatile phenols***										
4-vinylguaiacol	107.3	48.8	109.1	37.6	57.2	31.8	119.0	74.9	114.7	49.7
4-vinylphenol	72.6	35.2	101.8	9.7	50.5	11.5	70.4	5.9	73.3	6.9
***Fatty acids***										
Butanoic acid	29.3	8.3	33.5	11.3	28.4	17.3	24.3	8.7	33.3	21.9
2+3-methylbutyric acid	43.3	13.0	55.7	17.9	42.9	14.5	112.4	140.1	88.1	27.6
Hexanoic acid	1943.0 abc	432.3	2224.5 ab	492.7	1366.4 c	270.6	1675.9 bc	530.6	2382.5 a	535.8
Octanoic acid	8862.0 ab	1144.0	9663.5 a	1339.0	7032.7 b	1133.2	8351.4 ab	2434.2	10 532.7 a	2235.8
Decanoic acid	1602.0	1238.5	2063.2	1150.3	1362.0	841.2	1686.6	1269.2	2146.9	1372.5
***Lactones***										
γ-butyrolactone	162.3 b	47.0	161.5 b	86.6	91.2 b	38.1	160.9 b	50.9	253.0 a	65.6
***Carbonyl compounds***										
Acetoin	12.9	3.7	19.0	10.5	29.9	25.3	22.8	9.4	18.4	6.6

Parral; AC: Arch-Cane; VSP: Vertical Shoot-Positioned; SH: Scott-Henry and GDC: Geneva Double-Curtain Training systems. Different letters show significant differences among wines by Fisher’s least significant difference (LSD) at *p* < 0.05.

**Table 6:** Odor activity values (*OAV*) in Albariño wines from different training systems.

Compounds	Descriptor	Threshold (μg/L)	*OAV* > 1				
			Parral	AC	VSP	SH	GDC
2-phenylethanol	roses	10 000	0.8	1.0	0.6	1.0	1.5
Ethyl butanoate	fruity, strawberry	125	1.4	1.3	1.4	1.1	2.8
Ethyl hexanoate	fruity, strawberry	62	10.2	10.6	8.4	9.0	12.0
Ethyl octanoate	apple, sweetish	580	0.9	0.9	0.7	0.9	1.1
Isoamyl acetate	banana	30	115.3	92.0	107.7	74.2	86.6
2-phenylethyl acetate	rose, honey, tobacco	250	2.0	1.3	1.4	1.4	2.0
Linalool	floral, citrus	25	0.8	1.0	0.8	1.4	1.3
β-damascenone	baked apple	0.05	167.2	170.8	151.2	189.5	239.4
4-vinylguaiacol	clove, curry	40	2.7	2.7	1.4	3.0	2.9
2+3-methylbutyric acid	cheese, old hops, sweaty	34	1.3	1.6	1.3	3.3	2.6
Hexanoic acid	sweaty, cheese	420	4.6	5.3	3.3	4.0	5.7
Octanoic acid	sweaty, cheese	500	17.7	19.3	14.1	16.7	21.1
Decanoic acid	soapy, waxy	1000	1.6	2.1	1.4	1.7	2.2

Parral; AC: Arch-Cane; VSP: Vertical Shoot-Positioned; SH: Scott-Henry and GDC: Geneva Double-Curtain Training systems. Odour descriptors and odor threshold reported in the literature [1,2,28,29,41,42].
